# [1,2-Bis(2-pyridyl­meth­oxy)benzene-κ^4^ *N*,*O*,*O*′,*N*′]bis­(nitrato-κ*O*)copper(II)

**DOI:** 10.1107/S1600536810024773

**Published:** 2010-07-03

**Authors:** Ying-Hui Yu, Jin-Sheng Gao, Li-Xin Wang, Ying Liu, Guang-Feng Hou

**Affiliations:** a College of Chemistry and Materials Science, Heilongjiang University, Harbin 150080, People’s Republic of China; b Engineering Research Center of Pesticides of Heilongjiang Province, Heilongjiang University, Harbin 150080, People’s Republic of China; c Dalian Songliao Chemical Industry Corporation, Dalian 116031, People’s Republic of China

## Abstract

In the title compound, [Cu(NO_3_)_2_(C_18_H_16_N_2_O_2_)], the Cu^II^ ion is six-coordinated in a Jahn–Teller-distorted octa­hedral environment defined by two O and two N atoms from the ligand and two O atoms from two monodentate nitrate anions.

## Related literature

For the synthesis and general backround to flexible pyridyl-based ligands, see: Liu *et al.* (2010 ▶). For a related structure, see: Zhang *et al.* (2010 ▶).
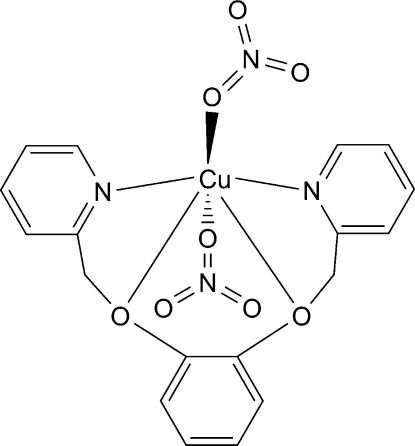


## Experimental

### 

#### Crystal data

[Cu(NO_3_)_2_(C_18_H_16_N_2_O_2_)]
*M* *_r_* = 479.90
Triclinic, 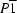

*a* = 8.621 (5) Å
*b* = 10.826 (6) Å
*c* = 10.887 (6) Å
α = 78.75 (2)°
β = 77.590 (19)°
γ = 76.54 (2)°
*V* = 953.8 (9) Å^3^
*Z* = 2
Mo *K*α radiation
μ = 1.20 mm^−1^
*T* = 291 K
0.31 × 0.30 × 0.19 mm

#### Data collection

Rigaku R-AXIS RAPID diffractometer
Absorption correction: multi-scan (*ABSCOR*; Higashi, 1995 ▶) *T*_min_ = 0.705, *T*_max_ = 0.808
9416 measured reflections
4314 independent reflections
3150 reflections with *I* > 2σ(*I*)
*R*_int_ = 0.039

#### Refinement

*R*[*F* ^2^ > 2σ(*F* ^2^)] = 0.046
*wR*(*F* ^2^) = 0.111
*S* = 1.04
4314 reflections
280 parameters
H-atom parameters constrained
Δρ_max_ = 0.45 e Å^−3^
Δρ_min_ = −0.40 e Å^−3^

### 

Data collection: *RAPID-AUTO* (Rigaku, 1998 ▶); cell refinement: *RAPID-AUTO*; data reduction: *CrystalClear* (Rigaku/MSC, 2002 ▶); program(s) used to solve structure: *SHELXS97* (Sheldrick, 2008 ▶); program(s) used to refine structure: *SHELXL97* (Sheldrick, 2008 ▶); molecular graphics: *SHELXTL* (Sheldrick, 2008 ▶); software used to prepare material for publication: *SHELXL97*.

## Supplementary Material

Crystal structure: contains datablocks I, global. DOI: 10.1107/S1600536810024773/ng2792sup1.cif

Structure factors: contains datablocks I. DOI: 10.1107/S1600536810024773/ng2792Isup2.hkl

Additional supplementary materials:  crystallographic information; 3D view; checkCIF report

## Figures and Tables

**Table 1:** Selected bond lengths (Å)

Cu1—O3	1.968 (2)
Cu1—O6	1.973 (2)
Cu1—N1	2.062 (3)
Cu1—N2	2.070 (2)
Cu1—O2	2.451 (3)
Cu1—O1	2.491 (2)

**Fig. 1.**
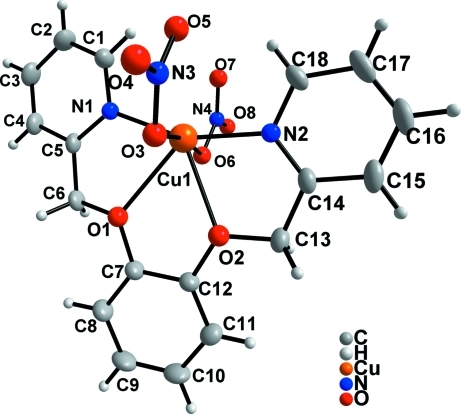
The molecular structure of title compound, showing the atom-labelling scheme and displacement ellipsoids drawn at the 30% probability level.

## supplementary crystallographic information

### Comment

In recent, our group has employed the flexible N-heterocyclic ligands reacting with transition metal to construct several supramolecular architectures (Liu *et al.* 2010; Zhang *et al.* 2010). As a part of our continuing work for bipyridyl aromatic ligands, we report the crystal s tructure of the title compound here.

1,2-Bis(pyridin-2-ylmethoxy)benzene molecule act as a chelating ligand to coordinate with Cu^II^ ion forming a discrete strucutre. Two nitrate anions also coordinate to the center Cu^II^ ion, resulting the Cu^II^ ion is six-coordinated in a typically Jahn-Teller distorted octahedral environment. Furthermore, a weak Cu—O bond, with distances of 2.742 (3) Å, between the Cu^II^ center and one nitrate anion link the coordination geometry into a distorted monocapped octahedron (Figure 1, Table 1).

### Experimental

The 1,2-Bis(pyridin-2-ylmethoxy)benzene was synthesized by the reaction of ο-dihydroxybenzene and 2-chloromethylpyridine hydrochloride under nitrogen atmosphere and alkaline condition (Liu *et al.*, 2010). Title ligand (0.58 g, 2 mmol) and Cu(NO_3_)_2_^.^H_2_O (0.48 g, 2 mmol) were dissolved in 15 ml e thanol, and then the mixture keep stirring for 30 minute. The resulting solution was filtered, and the filtrate was allowed to stand in a desiccator at room temperature for several days. Bule block crystals were obtained.

### Refinement

H atoms bound to C atoms were placed in calculated positions and treated as riding on their parent atoms, with C—H = 0.93 Å (aromatic C), C—H = 0.97 Å (methene C), and with *U*_iso_(H) = *1.2U*_eq_(C).

### Crystal data

**Table:**

[Cu(NO_3_)_2_(C_18_H_16_N_2_O_2_)]	*Z* = 2
*M**_r_* = 479.90	*F*(000) = 490
Triclinic, *P*1	*D*_x_ = 1.671 Mg m^−^^3^
Hall symbol: -P 1	Mo *K*α radiation, λ = 0.71073 Å
*a* = 8.621 (5) Å	Cell parameters from 6858 reflections
*b* = 10.826 (6) Å	θ = 3.4–27.5°
*c* = 10.887 (6) Å	µ = 1.20 mm^−^^1^
α = 78.75 (2)°	*T* = 291 K
β = 77.590 (19)°	Block, blue
γ = 76.54 (2)°	0.31 × 0.30 × 0.19 mm
*V* = 953.8 (9) Å^3^	

### Data collection

**Table:**

Rigaku R-AXIS RAPID diffractometer	4314 independent reflections
Radiation source: fine-focus sealed tube	3150 reflections with *I* > 2σ(*I*)
graphite	*R*_int_ = 0.039
ω scan	θ_max_ = 27.5°, θ_min_ = 3.4°
Absorption correction: multi-scan (*ABSCOR*; Higashi, 1995)	*h* = −11→10
*T*_min_ = 0.705, *T*_max_ = 0.808	*k* = −13→14
9416 measured reflections	*l* = −14→14

### Refinement

**Table:**

Refinement on *F*^2^	Primary atom site location: structure-invariant direct methods
Least-squares matrix: full	Secondary atom site location: difference Fourier map
*R*[*F*^2^ > 2σ(*F*^2^)] = 0.046	Hydrogen site location: inferred from neighbouring sites
*wR*(*F*^2^) = 0.111	H-atom parameters constrained
*S* = 1.04	*w* = 1/[σ^2^(*F*_o_^2^) + (0.0521*P*)^2^ + 0.2381*P*] where *P* = (*F*_o_^2^ + 2*F*_c_^2^)/3
4314 reflections	(Δ/σ)_max_ = 0.001
280 parameters	Δρ_max_ = 0.45 e Å^−^^3^
0 restraints	Δρ_min_ = −0.40 e Å^−^^3^

### Special details

**Table:**

Geometry. All e.s.d.'s (except the e.s.d. in the dihedral angle between two l.s. planes) are estimated using the full covariance matrix. The cell e.s.d.'s are taken into account individually in the estimation of e.s.d.'s in distances, angles and torsion angles; correlations between e.s.d.'s in cell parameters are only used when they are defined by crystal symmetry. An approximate (isotropic) treatment of cell e.s.d.'s is used for estimating e.s.d.'s involving l.s. planes.
Refinement. Refinement of *F*^2^ against ALL reflections. The weighted *R*-factor *wR* and goodness of fit *S* are based on *F*^2^, conventional *R*-factors *R* are based on *F*, with *F* set to zero for negative *F*^2^. The threshold expression of *F*^2^ > σ(*F*^2^) is used only for calculating *R*-factors(gt) *etc*. and is not relevant to the choice of reflections for refinement. *R*-factors based on *F*^2^ are statistically about twice as large as those based on *F*, and *R*- factors based on ALL data will be even larger.

### Fractional atomic coordinates and isotropic or equivalent isotropic displacement parameters (Å^2^)

**Table:**

	*x*	*y*	*z*	*U*_iso_*/*U*_eq_	
C1	0.5717 (4)	0.6307 (3)	0.2015 (3)	0.0476 (8)	
H1	0.5016	0.5749	0.2394	0.057*	
C2	0.7258 (4)	0.5802 (4)	0.1445 (3)	0.0562 (9)	
H2	0.7589	0.4923	0.1434	0.067*	
C3	0.8307 (4)	0.6624 (4)	0.0890 (3)	0.0551 (9)	
H3	0.9350	0.6308	0.0486	0.066*	
C4	0.7797 (4)	0.7903 (4)	0.0941 (3)	0.0483 (8)	
H4	0.8498	0.8465	0.0582	0.058*	
C5	0.6229 (3)	0.8369 (3)	0.1529 (3)	0.0379 (7)	
C6	0.5702 (3)	0.9769 (3)	0.1585 (3)	0.0437 (7)	
H6A	0.6479	1.0057	0.1933	0.052*	
H6B	0.5667	1.0237	0.0732	0.052*	
C7	0.3364 (4)	1.1288 (3)	0.2277 (3)	0.0438 (7)	
C8	0.4092 (4)	1.2346 (3)	0.1885 (3)	0.0523 (8)	
H8	0.5211	1.2240	0.1634	0.063*	
C9	0.3133 (5)	1.3562 (4)	0.1871 (4)	0.0620 (10)	
H9	0.3610	1.4279	0.1607	0.074*	
C10	0.1485 (5)	1.3722 (4)	0.2244 (3)	0.0586 (9)	
H10	0.0852	1.4546	0.2224	0.070*	
C11	0.0755 (4)	1.2666 (3)	0.2649 (3)	0.0490 (8)	
H11	−0.0363	1.2776	0.2898	0.059*	
C12	0.1699 (4)	1.1457 (3)	0.2678 (3)	0.0424 (7)	
C13	−0.0517 (4)	1.0379 (3)	0.3337 (3)	0.0481 (8)	
H13A	−0.0955	1.0785	0.2574	0.058*	
H13B	−0.1040	1.0888	0.4009	0.058*	
C14	−0.0837 (3)	0.9041 (3)	0.3722 (3)	0.0407 (7)	
C15	−0.2393 (4)	0.8881 (4)	0.4305 (3)	0.0555 (10)	
H15	−0.3201	0.9587	0.4495	0.067*	
C16	−0.2722 (4)	0.7667 (5)	0.4595 (3)	0.0633 (11)	
H16	−0.3756	0.7541	0.4984	0.076*	
C17	−0.1513 (5)	0.6645 (4)	0.4307 (3)	0.0626 (11)	
H17	−0.1724	0.5819	0.4475	0.075*	
C18	0.0027 (4)	0.6850 (4)	0.3761 (3)	0.0486 (8)	
H18	0.0850	0.6147	0.3588	0.058*	
Cu1	0.27619 (4)	0.81850 (4)	0.27623 (3)	0.03697 (13)	
N1	0.5180 (3)	0.7571 (3)	0.2049 (2)	0.0391 (6)	
N2	0.0374 (3)	0.8036 (3)	0.3473 (2)	0.0399 (6)	
N3	0.3327 (3)	0.6879 (3)	0.5135 (3)	0.0522 (7)	
N4	0.2099 (3)	0.7793 (3)	0.0525 (3)	0.0485 (7)	
O1	0.4174 (3)	1.0028 (2)	0.2342 (2)	0.0579 (7)	
O2	0.1161 (3)	1.0317 (2)	0.3109 (2)	0.0541 (6)	
O3	0.3212 (3)	0.8020 (2)	0.4491 (2)	0.0479 (5)	
O4	0.3539 (4)	0.6751 (3)	0.6242 (2)	0.0797 (9)	
O5	0.3246 (3)	0.5986 (3)	0.4623 (3)	0.0697 (7)	
O6	0.2278 (3)	0.8736 (2)	0.1024 (2)	0.0483 (5)	
O7	0.2304 (3)	0.6710 (3)	0.1141 (2)	0.0646 (7)	
O8	0.1728 (3)	0.8034 (3)	−0.0531 (2)	0.0794 (9)	

### Atomic displacement parameters (Å^2^)

**Table:**

	*U*^11^	*U*^22^	*U*^33^	*U*^12^	*U*^13^	*U*^23^
C1	0.0415 (17)	0.044 (2)	0.0546 (19)	−0.0102 (15)	−0.0042 (15)	−0.0046 (15)
C2	0.049 (2)	0.052 (2)	0.062 (2)	0.0012 (16)	−0.0054 (17)	−0.0134 (17)
C3	0.0343 (17)	0.074 (3)	0.0494 (19)	−0.0028 (17)	0.0021 (15)	−0.0115 (18)
C4	0.0292 (15)	0.064 (2)	0.0468 (17)	−0.0116 (15)	−0.0014 (14)	0.0001 (16)
C5	0.0279 (14)	0.0487 (19)	0.0345 (14)	−0.0105 (13)	−0.0050 (12)	0.0028 (13)
C6	0.0327 (15)	0.050 (2)	0.0473 (17)	−0.0182 (14)	−0.0010 (14)	−0.0005 (14)
C7	0.0417 (16)	0.0371 (18)	0.0511 (18)	−0.0123 (14)	−0.0040 (14)	−0.0024 (14)
C8	0.0495 (19)	0.047 (2)	0.060 (2)	−0.0182 (16)	−0.0063 (16)	−0.0008 (16)
C9	0.078 (3)	0.040 (2)	0.075 (2)	−0.0217 (19)	−0.018 (2)	−0.0074 (18)
C10	0.078 (3)	0.0350 (19)	0.064 (2)	−0.0062 (18)	−0.020 (2)	−0.0064 (16)
C11	0.0487 (19)	0.047 (2)	0.0480 (18)	−0.0010 (15)	−0.0106 (15)	−0.0081 (15)
C12	0.0436 (17)	0.0357 (17)	0.0473 (17)	−0.0096 (14)	−0.0070 (14)	−0.0036 (13)
C13	0.0330 (16)	0.056 (2)	0.0500 (18)	−0.0058 (14)	−0.0041 (14)	−0.0025 (16)
C14	0.0326 (15)	0.059 (2)	0.0325 (14)	−0.0162 (15)	−0.0063 (12)	−0.0020 (14)
C15	0.0326 (16)	0.091 (3)	0.0423 (17)	−0.0185 (18)	−0.0011 (14)	−0.0079 (18)
C16	0.0415 (19)	0.107 (3)	0.049 (2)	−0.042 (2)	−0.0039 (16)	0.001 (2)
C17	0.066 (2)	0.088 (3)	0.0475 (19)	−0.054 (2)	−0.0157 (18)	0.0114 (19)
C18	0.0539 (19)	0.056 (2)	0.0432 (17)	−0.0319 (17)	−0.0090 (15)	0.0012 (15)
Cu1	0.02889 (19)	0.0399 (2)	0.0400 (2)	−0.01305 (15)	0.00069 (14)	−0.00118 (15)
N1	0.0303 (12)	0.0465 (16)	0.0378 (13)	−0.0102 (11)	−0.0027 (10)	−0.0007 (11)
N2	0.0350 (13)	0.0520 (16)	0.0342 (12)	−0.0213 (12)	−0.0021 (11)	0.0005 (11)
N3	0.0310 (13)	0.065 (2)	0.0543 (17)	−0.0149 (13)	0.0011 (13)	0.0023 (15)
N4	0.0308 (13)	0.071 (2)	0.0424 (15)	−0.0182 (14)	−0.0001 (12)	−0.0039 (15)
O1	0.0366 (12)	0.0384 (13)	0.0859 (17)	−0.0129 (10)	0.0161 (12)	−0.0025 (12)
O2	0.0341 (11)	0.0381 (13)	0.0834 (17)	−0.0103 (10)	0.0038 (11)	−0.0055 (12)
O3	0.0424 (12)	0.0532 (15)	0.0473 (12)	−0.0145 (11)	−0.0035 (10)	−0.0044 (11)
O4	0.0758 (19)	0.110 (3)	0.0458 (15)	−0.0190 (18)	−0.0146 (14)	0.0102 (15)
O5	0.0650 (17)	0.0577 (18)	0.0864 (19)	−0.0236 (14)	−0.0116 (15)	0.0006 (15)
O6	0.0458 (12)	0.0476 (14)	0.0471 (12)	−0.0159 (11)	−0.0040 (10)	0.0069 (10)
O7	0.0703 (17)	0.0573 (17)	0.0655 (16)	−0.0189 (14)	−0.0083 (13)	−0.0044 (14)
O8	0.0679 (18)	0.134 (3)	0.0429 (14)	−0.0424 (19)	−0.0161 (13)	0.0037 (15)

### Geometric parameters (Å, °)

**Table:**

C1—N1	1.344 (4)	C13—C14	1.500 (5)
C1—C2	1.374 (4)	C13—H13A	0.9700
C1—H1	0.9300	C13—H13B	0.9700
C2—C3	1.380 (5)	C14—N2	1.346 (4)
C2—H2	0.9300	C14—C15	1.390 (4)
C3—C4	1.359 (5)	C15—C16	1.371 (6)
C3—H3	0.9300	C15—H15	0.9300
C4—C5	1.389 (4)	C16—C17	1.366 (6)
C4—H4	0.9300	C16—H16	0.9300
C5—N1	1.355 (4)	C17—C18	1.383 (4)
C5—C6	1.487 (5)	C17—H17	0.9300
C6—O1	1.395 (3)	C18—N2	1.348 (4)
C6—H6A	0.9700	C18—H18	0.9300
C6—H6B	0.9700	Cu1—O3	1.968 (2)
C7—O1	1.376 (4)	Cu1—O6	1.973 (2)
C7—C8	1.385 (4)	Cu1—N1	2.062 (3)
C7—C12	1.387 (4)	Cu1—N2	2.070 (2)
C8—C9	1.381 (5)	Cu1—O2	2.451 (3)
C8—H8	0.9300	Cu1—O1	2.491 (2)
C9—C10	1.371 (5)	Cu1—O7	2.742 (3)
C9—H9	0.9300	N3—O5	1.229 (4)
C10—C11	1.384 (5)	N3—O4	1.233 (4)
C10—H10	0.9300	N3—O3	1.290 (4)
C11—C12	1.370 (5)	N4—O8	1.223 (4)
C11—H11	0.9300	N4—O7	1.226 (4)
C12—O2	1.380 (4)	N4—O6	1.298 (4)
C13—O2	1.403 (4)		
			
N1—C1—C2	122.8 (3)	C17—C16—C15	119.2 (3)
N1—C1—H1	118.6	C17—C16—H16	120.4
C2—C1—H1	118.6	C15—C16—H16	120.4
C1—C2—C3	118.8 (3)	C16—C17—C18	119.5 (4)
C1—C2—H2	120.6	C16—C17—H17	120.3
C3—C2—H2	120.6	C18—C17—H17	120.3
C4—C3—C2	119.2 (3)	N2—C18—C17	122.1 (4)
C4—C3—H3	120.4	N2—C18—H18	119.0
C2—C3—H3	120.4	C17—C18—H18	119.0
C3—C4—C5	120.0 (3)	O3—Cu1—O6	168.05 (10)
C3—C4—H4	120.0	O3—Cu1—N1	91.68 (10)
C5—C4—H4	120.0	O6—Cu1—N1	90.92 (10)
N1—C5—C4	121.1 (3)	O3—Cu1—N2	91.37 (9)
N1—C5—C6	119.8 (3)	O6—Cu1—N2	90.77 (10)
C4—C5—C6	119.1 (3)	N1—Cu1—N2	157.09 (11)
O1—C6—C5	110.5 (2)	O3—Cu1—O2	85.99 (10)
O1—C6—H6A	109.5	O6—Cu1—O2	83.59 (10)
C5—C6—H6A	109.5	N1—Cu1—O2	131.82 (9)
O1—C6—H6B	109.5	N2—Cu1—O2	71.06 (9)
C5—C6—H6B	109.5	O3—Cu1—O1	83.11 (9)
H6A—C6—H6B	108.1	O6—Cu1—O1	86.74 (9)
O1—C7—C8	125.0 (3)	N1—Cu1—O1	70.71 (9)
O1—C7—C12	114.9 (3)	N2—Cu1—O1	132.20 (9)
C8—C7—C12	120.0 (3)	O2—Cu1—O1	61.22 (8)
C9—C8—C7	119.0 (3)	O3—Cu1—O7	140.28 (9)
C9—C8—H8	120.5	O6—Cu1—O7	51.65 (10)
C7—C8—H8	120.5	N1—Cu1—O7	83.49 (10)
C10—C9—C8	120.6 (3)	N2—Cu1—O7	79.70 (9)
C10—C9—H9	119.7	O2—Cu1—O7	125.77 (9)
C8—C9—H9	119.7	O1—Cu1—O7	130.79 (8)
C9—C10—C11	120.5 (4)	C1—N1—C5	118.1 (3)
C9—C10—H10	119.8	C1—N1—Cu1	117.6 (2)
C11—C10—H10	119.8	C5—N1—Cu1	124.1 (2)
C12—C11—C10	119.3 (3)	C14—N2—C18	118.0 (3)
C12—C11—H11	120.4	C14—N2—Cu1	124.1 (2)
C10—C11—H11	120.4	C18—N2—Cu1	117.8 (2)
C11—C12—O2	126.0 (3)	O5—N3—O4	123.7 (3)
C11—C12—C7	120.5 (3)	O5—N3—O3	119.2 (3)
O2—C12—C7	113.4 (3)	O4—N3—O3	117.0 (3)
O2—C13—C14	108.8 (3)	O8—N4—O7	123.4 (3)
O2—C13—H13A	109.9	O8—N4—O6	118.3 (3)
C14—C13—H13A	109.9	O7—N4—O6	118.3 (3)
O2—C13—H13B	109.9	C7—O1—C6	117.7 (2)
C14—C13—H13B	109.9	C7—O1—Cu1	123.01 (18)
H13A—C13—H13B	108.3	C6—O1—Cu1	112.23 (19)
N2—C14—C15	122.0 (3)	C12—O2—C13	117.3 (3)
N2—C14—C13	119.4 (2)	C12—O2—Cu1	124.99 (18)
C15—C14—C13	118.6 (3)	C13—O2—Cu1	113.57 (19)
C16—C15—C14	119.1 (4)	N3—O3—Cu1	115.3 (2)
C16—C15—H15	120.4	N4—O6—Cu1	112.66 (19)
C14—C15—H15	120.4	N4—O7—Cu1	77.37 (19)

### Hydrogen-bond geometry (Å, °)

**Table:**

*D*—H···*A*	*D*—H	H···*A*	*D*···*A*	*D*—H···*A*
C6—H6B···O6^i^	0.97	2.58	3.333 (4)	135
C13—H13A···O8^ii^	0.97	2.48	3.444 (5)	172
C13—H13B···O3^iii^	0.97	2.45	3.370 (4)	159
C17—H17···O5^iv^	0.93	2.53	3.412 (5)	159

Symmetry codes: (i) −*x*+1, −*y*+2, −*z*; (ii) −*x*, −*y*+2, −*z*; (iii) −*x*, −*y*+2, −*z*+1; (iv) −*x*, −*y*+1, −*z*+1.
